# Major histocompatibility complex class I-related chain A/B (MICA/B) expression in tumor tissue and serum of pancreatic cancer: Role of uric acid accumulation in gemcitabine-induced MICA/B expression

**DOI:** 10.1186/1471-2407-11-194

**Published:** 2011-05-23

**Authors:** Xiulong Xu, Geetha S Rao, Veronika Groh, Thomas Spies, Paolo Gattuso, Howard L Kaufman, Janet Plate, Richard A Prinz

**Affiliations:** 1 Department of General Surgery, Rush University Medical Center, 1653 W. Congress Parkway, Chicago, IL 60612, USA; 2 Fred Hutchinson Cancer Research Center, 1100 Fairview Avenue N, Seattle, WA 98109, USA; 3 Department of Pathology, Rush University Medical Center, 1653 W. Congress Parkway, Chicago, IL 60612, USA; 4 Department of Medicine, Rush University Medical Center, 1653 W. Congress Parkway, Chicago, IL 60612, USA; 5 Department of Immunology/Microbiology, Rush University Medical Center, 1653 W. Congress Parkway, Chicago, IL 60612, USA; 6 Department of Surgery, NorthShore University Health System, Evanston, IL 60201, USA

**Keywords:** Pancreatic cancer, MICA/B, Gemcitabine, Uric acid, Allopurinol, DNA damage

## Abstract

**Background:**

Major histocompatibility complex class I-related chain A and B (MICA/B) are two stress-inducible ligands that bind the immunoreceptor NKG2D and play an important role in mediating the cyotoxicity of NK and T cells. In this study, we sought to study MICA/B expression in pancreatic cancer and to determine whether and how genotoxic drugs such as gemcitabine can affect MICA/B expression and natural killer cytotoxity.

**Methods:**

Seven pancreatic cancer cell lines were analyzed for MICA/B expression by flow cytometry and for their sensitivity to NK-92 cell killing by a ^51^Cr release assay. MICA/B expression in tumor tissues and sera of pancreatic cancer was analyzed by immunohistochemical staining (IHC) and ELISA, respectively.

**Results:**

Two MICA/B-positive cell lines were sensitive to the cytotoxic activity of NK-92 cells. Other two MICA/B-positive cell lines and three MICA/B-negative cell lines were resistant to NK-92 cell killing. MICA/B expression was positive in 17 of 25 (68%) pancreatic ductal adenocarcinomas but not in normal pancreatic ductal epithelial cells. Serum MICA/B levels were significantly elevated in patients with pancreatic adenocarcinomas but did not correlate with the stage of pancreatic cancer and patient survival. Gemcitabine therapy led to increased serum MICA levels in 6 of 10 patients with detectable serum MICA. Allopurinol, an inhibitor of xanthine oxidoreductase that converts xanthine to uric acid, blocked uric acid production, MICA/B expression, and sensitivity to NK-92 cell killing toward a PANC-1 cancer cell line exposed to radiation and two genotoxic drugs, gemcitabine and 5-fluorouracil.

**Conclusions:**

The levels of MICA/B expression in serum and tissue of pancreatic cancer are elevated. DNA damage-induced MICA/B expression is mediated through increased uric acid production.

## Background

Pancreatic cancer remains one of the most lethal human cancers and causes > 30,000 deaths per year in the United States [1]. Conventional treatments, such as surgery, radiation, and chemotherapy, or combination of these approaches, have had little impact on patient survival [1]. Over the past decade, improved understanding and knowledge of the immune system have generated novel strategies for immunotherapy [2]. While the success of tumor immunotherapy primarily relies on the identification of tumor antigens, the expression of transformation-associated stress genes commonly provokes innate immune reactions. These responses could be exploited to develop immunotherapeutic approaches to treat cancer [3].

MICA is a glycosylated, polymorphic and membrane-anchored non-classical MHC class I molecule [4,5]. The structure of MICA resembles other MHC class I heavy chains. However, MICA is not associated with β2 microglobulin, lacks a CD8 binding site and does not present any antigens [4,5]. MICA is broadly expressed in a variety of malignancies, including melanoma, breast, colon and hepatocellular cancers [6-8]. MICA can be cleaved by matrix metalloproteinases and ADAM proteinase, and released into the blood stream or tissue culture medium as a soluble molecule (sMICA) [7,9-11]. MICA functions as a ligand for NKG2D, an important immunoreceptor expressed on NK cells, CD8 and γδ T cells [5]. The interaction of MICA and NKG2D plays an important role in immune surveillance by both innate immunity and adaptive immunity. *In vitro *studies have provided strong evidence that MICA is critical for the susceptibility of target cells to NK cells, CD8 cytotoxic T cells, and γδ T cells [5]. Antibodies that block the interaction of MICA and NKG2D can inhibit NK and T cell-mediated cytolysis [5]. Tumor cells stably expressing NKG2D ligands at high level are rejected by CD8 T cells and/or NK cells [12]. Mice immunized with NKG2/D ligand-transfected tumor cells develop adaptive immunity against re-challenge with the parental tumor cell lines [13].

Gemcitabine is a first-line chemotherapy drug for pancreatic cancer [14]. Gemcitabine alone or in combination with 5-fluorouracil (5-FU) or radiation treatment can prolong the survival of pancreatic cancer patients [14]. We have recently characterized the change of immune cells in pancreatic cancer patients treated with gemcitabine [15]. Our data suggest that gemcitabine therapy may decrease memory T-cells and promote naive T-cell activation, and that gemcitabine therapy is not immunosuppressive but rather may enhance antitumor immunity induced by tumor vaccine [15]. Our present study aims at analyzing MICA/B expression in pancreatic tumor tissues and sera, and determining if gemcitabine can stimulate antitumor immunity by inducing MICA/B expression on pancreatic cancer cells.

## Materials and methods

### Reagents and cell lines

Anti-MICA/B mAbs (Clones 6D4 and SR99) have been previously described [6,16-18]. Both antibodies can be used in flow cytometric analysis to detect MICA/B cell surface expression. SR99 but not 6D4 can be used in immunohistochemical staining to monitor MICA/B expression in sections of paraffin-embedded tumor blocks. A polyclonal anti-MICA rabbit IgG was purchased from Santa Cruz Biotechnology, Inc. (Santa Cruz, CA). CAPAN-1, CAPAN-2, COLO-587, HPAF-II, MiaPaCa, PANC-1, and MPANC-96 cell lines were purchased from the American Type Culture Collection (Manassas, VA). MRO87, originally thought to be a thyroid cancer cell line but later identified as a colon adenocarcinoma cell line, was included as a positive control [19]. CAPAN-1, CAPAN-2, and HPAF-II cells were grown in complete MEM medium containing 10% FBS, non-essential amino acids, sodium pyruvate, HEPES, penicillin and streptomycilin. COLO-587 and MRO87 cells were grown in complete RPMI 1640 medium containing 10% FBS. PANC-1 cells were grown in complete RPMI 1640 medium containing 10% FBS, non-essential amino acids, sodium pyruvate, HEPES, penicillin and streptomycilin. MPANC-96 and MiaPaCa were grown in complete DMEM medium containing 10% FBS. NK-92 cells were grown in MyeloCult H5100 medium (StemCell Technologies, Vancouver, British Columbia, Canada) supplemented with IL-2 (100 units/ml) (R&D Systems).

### Patient information, tumor specimens and blood samples

The use of specimens from human subjects was approved by the Institutional Review Board of Rush University Medical Center. Paraffin-embedded tissue blocks derived from patients with either primary pancreatic adenocarcinomas and/or metastases were obtained from the Department of Pathology. The stage of the pancreatic adenocarcinomas was classified according to the TNM scheme used by the AJCC. Blood samples were collected from 61 patients with pancreatic ductal adenocarcinoma. Ten of them were treated with gemcitabine (Gemzar^®^; Eli Lilly, Indianapolis, IN) reconstituted from lyophilized powder in 200-mg and 1000-mg aliquots. Patients were treated at a dose of 1000 mg/m^2 ^weekly by an intravenous infusion in 250 mL over 30 minutes. Patients were treated for 3 weeks. Blood samples were drawn before (day 0) and on day 3, 7, 14, 21 after gemcitabine treatment. Serum samples were prepared, aliquoted, and stored at -80°C until analysis of sMICA using a sandwich ELISA as described below.

### Flow cytometric analysis

The monolayers of MRO87 and 7 pancreatic cancer cell lines were harvested when they reached approximately 80% confluence. To determine whether DNA damage stimulated MICA/B expression in pancreatic cancer cells, PANC-1 cells grown in 6-well plates were preincubated with allopurinol (250 μg/ml) for 1 hr, followed by treatment with 5-FU (10 μM), gemcitabine (2 μM) or radiation (40 Gy). PANC-1 cells were also treated with uric acid crystals (200 μg/ml) and analyzed for MICA/B expression. Cell surface MICA/B was stained with an anti-MICA/B mAb (Clone 6D4) and analyzed on live cells, which were gated on the forward scattering, in a FACScan flow cytometer (Becton Dickinson, Palo Alto, CA) as previous described [20].

### Chromium release assay

Pancreatic cancer cell lines were grown in T-25 flasks. Upon 90% confluence, the cell monolayers were washed and treated with Cell Dissociation Solution (Sigma, St. Louis, MO). Single cell suspensions were labeled with ^51^Cr ( 50 μ Ci per 1 × 10^6 ^cells) at 37°C for 1 hr. The cells (5000 per well) were aliquoted in triplicate in a 96-well U-bottom plate in the absence or presence of NK-92 cells with a ratio of effector to target at 25:1, 5:1, or 1:1. Cells were incubated at 37°C in a humidified CO_2 _incubator for 4 hr. The supernatants were collected, transferred to Ready-Caps, and the radioactivity was measured in a scintillation counter (Becton Dickinson, Palo Alto, CA). To determine whether genotoxic drugs and radiation can sensitize pancreatic cancer cells for NK-92 cell killing, PANC-1 cells grown in 6-well plates were preincubated with 5-FU (10 μM), gemcitabine (2 μM) or radiation (40 Gy) in the absence or presence of allopurinol (250 μg/ml). The experiments were conducted in triplicate and repeated at least twice with similar results.

### Immunohistochemical (IHC) analysis

Tissue sections were de-paraffinized with xylene and rehydrated. MICA/B were stained with an anti-MICA/B mAb (SR99) as previously described [20]. Two investigators (X.X. and P. G.) graded MICA/B expression in a blinded fashion. MICA/B expression was confirmed with a rabbit polyclonal anti-MICA/B IgG with a similar immunohistochemical staining procedure as previously published [20]. Negative MICA/B expression was graded as no MICA/B signal (-)with weak signal (+) in less than 20% of tumor cells. MICA/B-positive tumors were defined as weak (+), moderate (++) or strong (+++) MICA/B signal in more than 20% of tumor cells.

### ELISA

A MICA ELISA kit (IMMATICS Biotechnologies, Tubigen, Germany) was used to detect sMICA in sera following a protocol described previously [21]. Plates were coated with the AMO1 capture mAb against MICA in PBS. After incubation overnight at 4°C, the plates were blocked with PBS containing 5% bovine serum albumin for 1 hr at 37°C and washed. Standard recombinant sMICA and serum samples (diluted 1:3) were added to the plates and incubated for 2 hrs at 37°C. After 3x wash, detection mAb BAMO3 (IgG2a specific for MICA/B) was added and incubated at 37°C for 2 hrs. The plates were washed and incubated at 37°C with HRP- conjugated anti mouse IgG2a. Color was developed using ABTS (2,2-Azino-bis-(3-ethylbenzenthiazoline-6-sulfnoic acid). Absorbance was measured at 450 nm. A standard curve of the logarithimic relationship between concentration and absorbance was used to calculate the sMICA concentration in serum samples.

### Quantification of uric acid

PANC-1 cells were left untreated or treated with 5-FU (10 μM), gemcitabine (2 μM) or irradiated with 40 G in the absence or presence of allopurinol (250 μg/ml) for 24 hr. Cells were collected by trypsinization and counted with Trypan Blue staining in a hemacytometer. The cells were then washed 3 times with PBS and lysed at 5 × 10^5 ^cells/100 μl in the buffer containing Tris-HCl, 50 mM, pH 8.0; 2 mM EDTA, 1% Triton X-100 and followed by a 10-second homogenization. The lysates were incubated on ice for 30 min then centrifuged at 2000 g for 15 min. The supernatants were analyzed for uric acid concentration by using a uric acid kit (BioAssay Systems, Hayward, CA).

### Statistical analysis

Chi-square tests were performed to analyze the difference in the clinicopathologic parameters of MICA/B-positive and MICA/B-negative pancreatic ductal adenocarcinomas for significant association. Student *t *test was used to analyze the difference in NK-92 cell-mediated cytotoxicity. Mann-Whitney *U *test was used to compare serum MICA levels between the control group and pancreatic cancer patients. Paired student *t *test was used to determine whether serum MICA/B levels were significantly changed before (day 0) and on day 3 after gemcitabine treatment. The survival of pancreatic cancer patients with serum MICA/B activity at a cutoff of 200 pg/ml was determined according to the Kaplan-Meier method, and the difference was evaluated by the log-rank test. A *p *value less than 0.05 was considered statistically significant. All statistics was conducted by using SigmaStat 3 software (Richmond, CA).

## Results

### MICA/B expression in pancreatic cancer cell lines

**Figure 1:**
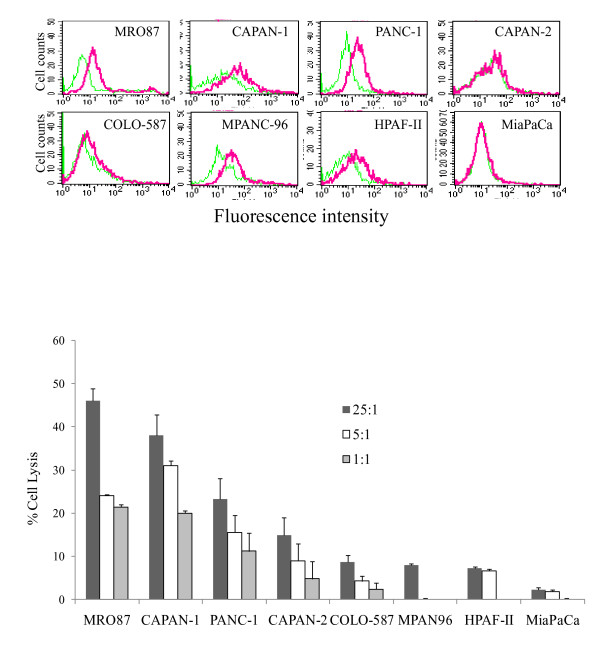
**MICA/B expression in pancreatic cancer cell lines and their sensitivity to NK-92 cell-mediated cytotoxic activity**. (**A**) FACS analysis of MICA/B expression in MRO87 and 7 pancreatic tumor cell lines. Cells (1 × 10^6^/sample) were stained with 1 μg of anti-MCA/B mAb (clone 6D4) followed by 10 μl of fluorescein-conjugated goat-anti-mouse IgG. Isotype-matched mouse IgG was used as a negative control. Cells were analyzed in a BD FACScan flow cytometer. Green line, IgG control; Red line, anti-MICA/B mAb. (**B**) Cytotoxicity of NK-92 cells against 8 tumor cell lines. Single cell suspensions were prepared and analyzed for cytolysis by NK-92 cells in a standard ^51^Cr release assay. Experiments were conducted in triplicate and repeated at least twice. The data represent the mean ± standard deviation (SD) from one representative experiment.

MICA/B expression was analyzed in 7 pancreatic cancer cell lines by flow cytometry. MICA/B expression was detected in a positive control cell line (MRO87) and in 4 pancreatic cancer cell lines (PANC-1, MPANC96, HPAF-II, and CAPAN-1) but not in the other 3 cell lines (COLO-587, MiaPaCa, and CAPAN-2) (Figure 1A). Next, MICA/B expression was correlated with their sensitivity to cytotoxic activity mediated by NK-92 cells, a natural killer cell line that expresses NKG2D receptor [20]. Three MICA/B-negative cell lines (COLO-587, MiaPaCa, and CAPAN-2) were not sensitive to NK-92 cell killing (Figure 1B). Percent cytolysis of these cell lines by NK-92 cells assayed with a ratio of effector to target cells of 5:1 or 1:1 were less than 10%. Two of four MICA/B-positive cell lines, CAPAN-1 and PANC-1, were sensitive to NK-92 cell-mediated killing, with a percent cytolysis of approximately 20% when the ratio of effector to target cells was 5:1 or 1:1. Two MICA/B-positive cell lines, MPANC96 and CAPAN-1, were resistant to NK-92 cell killing.

### MICA/B expression in pancreatic ductal adenocarcinomas

**Figure 2:**
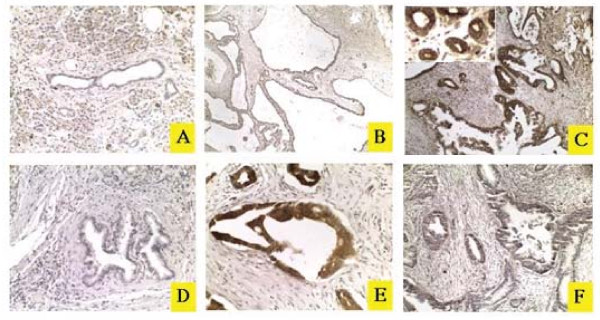
**MICA/B expression in pancreatic cancer by IHC analysis**. The sections of pancreatic adenocarcinomas were analyzed for MICA/B expression by immunohistochemical staining with a monoclonal antibody (Clone SR99) (30 μg/ml) (A-C) or a polyclonal anti-MICA rabbit IgG (1:60) (E-F). Lack of MICA/B expression in normal pancreatic ductal epithelial cells (A) and a cyst adenocarcinoma (B). Mouse IgG isotype was included as a negative control (D). (C & E) MICA/B signals in the membrane and cytoplasm. (F) No signal was present in a MICA/B-negative pancreatic cancer.

Twenty-five pancreatic cancer specimens from 25 patients (15 male and 10 female) with a mean age of 65 ± 11 (median age: 65, range: 38-81 years) were analyzed for MICA/B expression by immunohistochemical staining according our previous publication [18]. These 25 samples included 23 primary adenocarcinomas and 3 metastatic implants (one in the peritoneum, one in the liver, and one in the gallbladder). IHC staining with an anti-MICA/B mAb (SR99) (Figure 2,A-C) revealed the strong dark brown signal of MICA/B expression present in the tumor cells (Figure 2C) but not in normal ductal cells (Figure 2A) nor in a cyst adenoma (Figure 2B). Negative control IgG did not show any signal (Figure 2D). MICA/B expression was confirmed with a polyclonal anti-MICA/B antibody (Figure 2E). A MICA/B-negative pancreatic adenocarcinoma is shown in Figure 2F. Eighteen of (68%) of 25 pancreatic adenocarcinomas were positive for MICA/B (Table 1). Among these MICA/B-positive tumors, MICA/B expression was graded as weak, moderate, and strong in 2, 5, 11 samples, respectively. Clinicopathologic analyses did not reveal that MICA/B expression was associated with patient gender, tumor stage, and lymph node metastasis, and differentiation (Table 1).

**Table 1:** MICA/B expression in 25 pancreatic cancers and clinicopatholgoic significance

Gender	Total	MICA/B+	*p *value
Female	10	7	
Male	15	11	0.66
**Tumor stage**			
T1	3	1	
T2	9	7	
T3	8	6	> 0.05
T4	1	1	
Metastases	3	3	
**Lymph node invasion**			
Yes	14	10	
No	8	5	1.00
**Tumor differenation**			
Poorly differentiated	3	2	
Well differentiated	22	16	0.47

### Increased serum MICA levels in pancreatic cancer patients

The median and mean serum MICA levels in 61 patients were 228 pg/ml and 1107 ± 46 pg/ml respectively. The median and mean sMICA concentrations in 26 healthy control subjects were 30 and 211 ± 18 pg/ml, respectively (Table 2). Statistical analyses revealed that serum MICA levels were significantly increased in pancreatic cancer patients, compared to those in the healthy controls (*p *= 0.002) (Table 2).

**Table 2:** Increased serum sMICA levels and its clinicopathologic significance

	Number	Median	Mean ± SE	*p *value
Healthy donors	26	30	211 ± 18	
Pancreatic cancer patients	61	228	1107 ± 46	0.002
				
Female	25	220	461 ± 173	
Male	36	238	1556 ± 582	0.901
				
with surgery	24	145	390 ± 177	
without surgery	37	267	1572 ± 565	0.018
				
Stage I-III	15	144	437 ± 40	
Stage IV	46	231	1325 ± 69	0.178

No difference in serum MICA levels amongst male and female patients was revealed. The mean and median serum MICA levels in 25 female patients were 220 and 461 ± 173 pg/ml respectively; whereas the mean and median serum MICA levels in 36 male patients were 238 and 1556.2 ± 582 pg/ml, respectively (Table 2). Mann-Whitney test revealed that there was no significant difference in serum MICA levels between female and male patients (*p *= 0.901). The association between serum MICA levels with different tumor stages was also determined. The median and mean serum MICA levels in 15 patients with tumor stages I-III were 144 and 437 ± 40 pg/ml respectively, compared to 231 and 1325 ± 69 pg/ml in 46 patients with a stage IV tumor (Table 2). Statistical analyses revealed that MICA levels in patients with tumor stages I-III were not significantly lower than those with a stage IV tumor (*p *= 0.178). Surgical resection is an option for patients with earlier stage disease, hence serum MICA levels in 24 patients who underwent surgery were lower than those who did not. The median and mean serum levels in 24 patients who underwent Whipple procedure (9 cases) or tumor resection (15 cases) were 145 and 390 ± 177 pg/respectively, whereas the median and mean serum levels in 37 patients (14 cases with palliative surgery and 23 cases without any surgery) were 267 and 1572 ± 565 pg/ml, respectively (Table 2). Mann-Whitney test revealed a *p *value of 0.018, indicating that serum MICA levels in patients who were eligible for tumor resecton is significantly lower than those with unresectable tumors.

**Figure 3:**
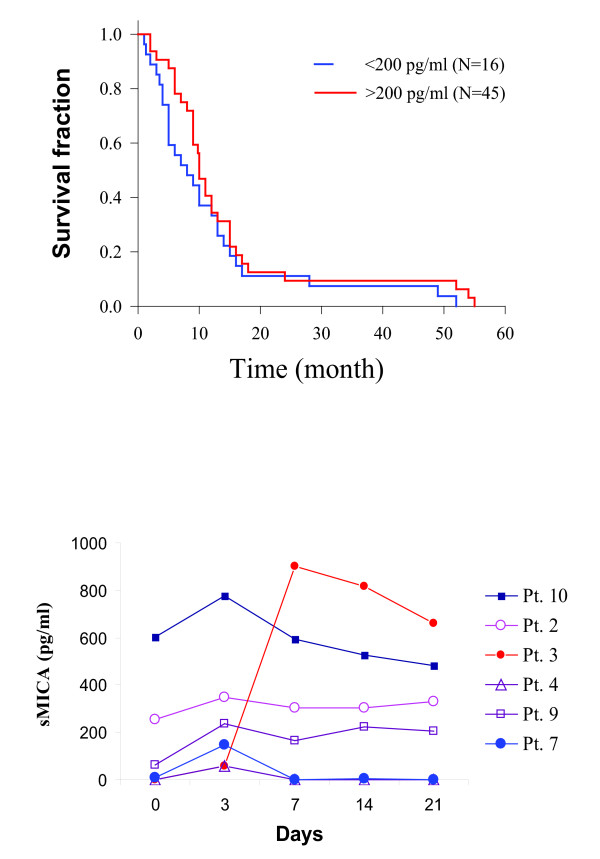
**Analysis of serum MICA levels in pancreatic cancer patients**. (**A**) Increased serum sMICA levels were not associated with patient survival. Kaplan-Meier survival curves in 16 patients with serum MICA levels < 200 pg/ml and in 45 patients with serum MICA levels > 200 pg/ml were compared. (**B**) Serum sMICA levels were elevated in pancreatic cancer patients treated with gemcitabine. Blood samples collected from 10 patients before (Day 0) and after gemcitabine treatment at the indicated days were analyzed for serum MICA levels using a sandwich ELISA kit. A standard curve of the logarithimic relationship between concentration and absorbance was used to calculate serum MICA concentrations. Data from four patients whose serum MICA levels were undetectable before and after gemcitabine treatment are not shown.

Pancreatic cancer patients were divided into two groups according to a cutoff value of 200 pg/ml of serum MICA levels. The mean survival of these 16 pancreatic cancer patients with serum MICA levels < 200 pg/ml was 10.3 ± 7.6 months; whereas the mean survival of 45 pancreatic cancer patients with serum MICA levels > 200 pg/ml was 10.3 ± 4.9 months (Figure 3A). Serum MICA/B levels was not associated with survival in pancreatic cancer patients (*p *= 0.233). We next tested whether gemcitabine treatment led to increased serum MICA levels in pancreatic cancer. Blood samples were taken from 10 patients before (day 0) and on day 3, 7, 14, and 28 after drug administration. Serum MICA levels were increased in 6 of 10 patients with detectable serum MICA levels (Figure 3B). Paired student *t *test analysis revealed that serum sMICA levels were significantly higher 3 days after gemcitabine treatment (day 0) than those before treatment (*p *= 0.011). Serum MICA levels in 4 patients with undetectable serum MICA level before gemcitabine administration remained undetectable during 4 weeks of gemcitabine treatment.

### Allopurinol blocks DNA damage-induced MICA/B expression, uric acid production, and sensitivity of PANC-1 cells to NK cell killing

**Figure 4:**
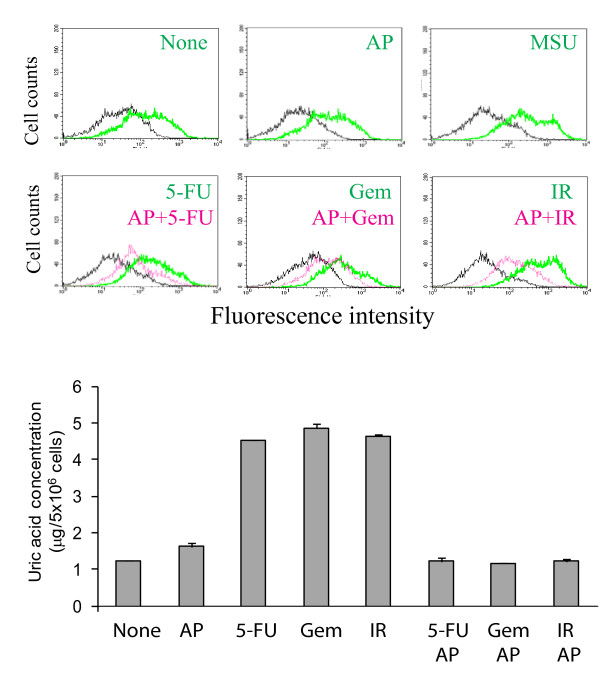
**Allopurinol blocks DNA damage-induced MICA/B expression**. PANC-1 cells were treated with uric acid crystals (250 μg/ml each), 5-FU (10 μM, gemcitabine (2 μM) or radiation (40 Gy) in the absence or presence of allopurinol (250 μg/ml). After incubation for 24 hr, the cells were harvested and analyzed for MICA/B expression by FACS (Black line, isotype control) (**A**) or for intracellular uric acid concentrations by using a colometric uric acid kit (**B**). The data represent the mean ± SD from one of two experiments with similar results. AP, allopurinol; MSU, uric acid crystals (monosodium urate); 5-FU, 5-fluorouracil; Gem, gemcitabine, IR, radiation.

The ability of gemcitabine and other DNA damage reagents to induce MICA/B expression was verified in vitro. Gemcitabine, 5-FU and radiation all led to increased cell surface MICA/B expression in PANC-1 cells (Figure 4A). Allopurinol, an inhibitor of xanthine oxidoreductase that converts xanthine to uric acid, was added to PANC-1 cells 1 hr prior to the addition of gemcitabine, 5-FU, or irradiation. Allopurinol was able to block the induction of MICA/B expression in the cells exposed to 5-FU, gemcitabine, or radiation (Figure 4A). To confirm a role of uric acid in mediating DNA damage-induced MICA/B expression, exogenous uric acid crystals were added and successfully induced MICA/B expression in PANC-1 cells (Figure 4A). Analysis of uric acid concentrations in PANC-1 cell lysates revealed that uric acid levels were increased by approximately 3-fold in PANC-1 cells treated with gemcitabine, 5-FU, and radiation (Figure 4B), compared to untreated controls. Allopurinol blocked the induction of uric acid accumulation (Figure 4B). Of note, Trypan blue staining revealed that 5-FU, gemcitabine, or radiation treatment during 24 hr incubation did not lead to a significant number of dead cells (< 5% of stained cells), suggesting that increased uric acid concentrations in DNA-damaged cells were not due to the variation caused by uncounted dead cells.

**Figure 5:**
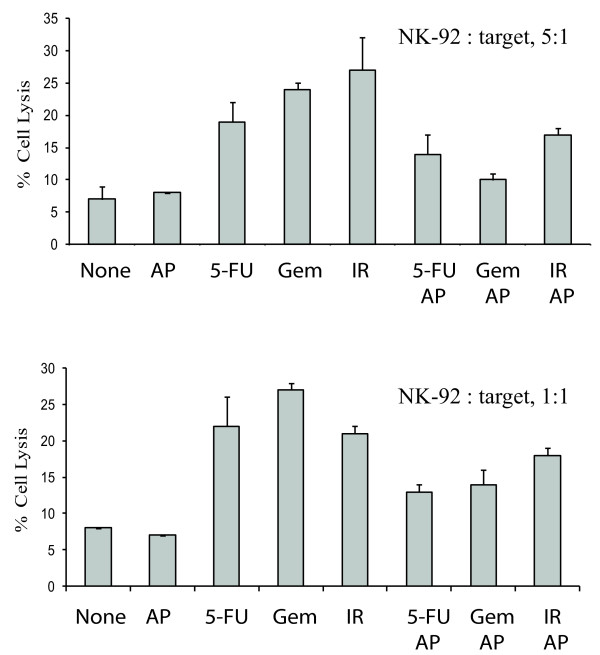
**Allopurinol blocks DNA damage-induced sensitization of PANC-1 cells to NK-92 cell killing**. PANC-1 cells grown in 35-mm dishes were left untreated or treated with 5-FU (10 μM), gemcitabine (2 μM) or irradiated (40 Gy) in the absence or presence of allopurinol (250 μg/ml). After incubation for 20 hr, the cells were harvested and analyzed using a standard ^51^Cr release assay for NK-92 cell cytotoxic activity with a ratio of effector:target cells at 5:1 (**A**) or 1:1 (**B**). The data represent the mean ± SD from one of two experiments with similar results. AP, allopurinol; 5-FU, 5-fluorouracil; Gem, gemcitabine, IR, radiation.

Pre-treatment of PANC-1 cells with gemcitabine, 5-FU, or radiation led to a significant increase of the percent ^51^Cr release (19-27%) in DNA-damaged cells, compared to that in untreated or allopurinol-treated cells (7-8%) (Figure 5A &5B). Allopurinol alone did not have any effect on the sensitivity of PANC-1 to NK-92 cell killing. However, allopuinol blocked DNA damaged-induced sensitization of PANC-1 cells to NK-92 cytotoxicity (Figure 5A &5B). These observations suggest that uric acid production and increased MICA/B expression play an important role in sensitizing pancreatic cancer cells to NK-92 cell-mediated cytotoxicity.

## Discussion

It has been well documented that MICA/B expression is increased in several types of malignancy. A very recent study showed that MICA/B expression was detected in 92 of 103 pancreatic ductal adenocarcinomas from a cohort of Chinese patients [22]. Marten et al. reported that MICA/B was expressed in three pancreatic cancer cell lines, including PANC-1, DNA-G, and PatSci [21]. Our study confirms the expression of MICA/B in pancreatic adenocarcinomas, showing that 17 of 25 pancreatic adenocarcinomas (68%) were positive for MICA/B expression; and that MICA/B expression was detected in 4 of 7 pancreatic cancer cell lines. The molecular mechanisms of increased MICA/B expression in pancreatic cancer are unknown. Our prior study suggests that MAP kinase activation due to BRAF gene mutation in thyroid cancer may contribute to increased MICA/B expression [20]. KRAS is mutated in more than 95% of pancreatic cancers [14]. We speculate that MAP kinase activation due to RAS gene mutation may in part contribute to increased MICA/B expression. Further epigenetic changes may be required for MICA/B overexpression since MICA/B promoter methylation regulates MICA/B gene expression in hepatomas [23].

Ectopic expression of NKG2D ligands Rae1β or H60 in several murine tumor cell lines leads to the sensitization of these cells to immune cell-mediated cytolysis and tumor rejection [13]. In contrast to murine NKG2D ligands, tumor-derived soluble MICA/B molecules can induce endocytosis and degradation of NKG2D on both tumor-infiltrating and peripheral-blood lymphocytes from patients with cancer [7,24]. Thus, serum MICA/B molecules released from tumor cells act as a negative force to counteract the effect of membrane-bound MICA/B in immune surveillance and sensitization for immune cell killing. Cell surface levels of NKG2D in NK and T cells from cancer patients are decreased, subsequently leading to the loss of cytotoxic activity [22,25]. Therefore, MICA/B expression in pancreatic cancer and subsequent release as soluble molecules may have a very intriguing impact in clinical outcome. In this study, we found that elevated serum MICA levels were not associated with a prolonged or shorter survival. In contrast, Duan et al. [22] recently reported that the mean survival of pancreatic cancer patients with low serum sMICA levels are significantly longer than those with high serum MICA levels, whereas the mean survival of patients with low MICA/B expression in pancreatic tumor tissues are significantly shorter than those with high MICA/B expression in tumor tissues. We have no explanation for this discrepancy. We found that serum sMICA levels in patients with stage IV pancreatic cancer were higher than those with stage I to III cancer, but this was not statistically significant. An earlier study showed that sMICA levels in patients with stage IV pancreatic cancer are significantly higher than those with stage III cancer [21].

NK-92 cells have a superior effect over lymphokine activated killer cells and other NK cell lines such as the YT cell line [26], largely because of an abundant expression of perforin and granzyme B [27]. Romanski et al. [28] showed that NK-92 cells can selectively kill a panel of leukemia cell lines in a MICA/B-dependent manner. NK-92 cells moderately express the NKG2D receptor and two other NK activation receptors, NKp30 and NKp46 [27]. NK-92 cells preferentially kill a panel of MICA/B-positive tumor cell lines [20]. In this study, we demonstrated that NK-92 cells were able to kill two MICA/B positive pancreatic cancer cell lines (PANC-1 & CAPAN-1) but had only minimal effect on other two MICA/B-positive cell lines (HPAF-II and MPANC-96). Marten et al. [21] found that NK-92 cells are able to kill HLA-ABC-positive PANC-1 cells with similar potency. The resistance of MICA/B-positive pancreatic cancer to NK-92 cell killing could be due to the inhibitory effect of co-expressed HLA-G antigen that suppresses NK cell function [29-31]. Three MICA/B-negative cell lines, CAPAN-2, COLO-587, and MiaPaCa, displayed very low to moderate sensitivity to NK-92 cells. It is likely that other NK activation receptors or other members of MICA/B such as ULBP1-4 and Letal, a recently identified NKG2D ligand [32,33], may also participate in NK-92 cell-mediated cytotoxic activity against MICA/B-negative tumor cell lines [21].

Activation of the DNA damage pathway leads to increased expression of NKG2D ligands in several murine tumor cell lines [34]. Other non-genotoxic anticancer drugs can also increase MICA/B and other NKG2D ligand expression in various tumor cell lines and in patients [35-37]. Mechanistic studies suggest that increased cell surface MICA/B levels by anticancer drugs could be due to transcriptional up-regulation of *MICA/B *gene expression or due to the suppression of proteinases that cleave MICA/B [35-37]. Our prior study showed that MAP kinase activation due to RAS or BRAF oncogene activation contributed to increased MICA/B expression [20]. Additional study showed that DNA damage induced by genotoxic drugs and radiation leads to uric acid accumulation and MAP kinase activation, subsequently resulting in increased MICA/B expression in MRO87 and HeLa cell lines (Manuscript under review). Our in vitro study showed that uric acid crystals were able to induce MICA/B expression in PANC-1 cells, and that the blockade of uric acid production by allopurinol abrogated DNA damage-induced uric acid production and MICA/B expression. Our clinical study showed that gemcitabine treatment led to a transient increase of serum sMICA levels in 6 of 10 pancreatic cancer patients. These observations collectively suggest that uric acid accumulation in DNA-damaged pancreatic cancer cells plays an important role in mediating genotoxic drug- and radiation-induced MICA/B expression. Consistent with this notion, we were able to detect the expression of xanthine oxidoreductase, a metabolic enzyme that generates uric acid, in PANC-1 cell line and in pancreatic adenocarcinomas (X. Xu, unpublished observations).

Since MICA/B expression in PANC-1 cells plays a critical role in NK-92 cell-mediated cytotoxicity [21], it is highly likely that increased MICA/B expression contributes significantly to DNA damage-enhanced sensitivity of PANC-1 cells to NK-92 cell killing. Increased MICA/B expression in HeLa and MRO87 cells by uric acid crystals also leads to increased sensitivity to NK-92 cell killing. It should be noted that other NKG2D ligands or Fas can be up-regulated by DNA damage too and may also sensitize tumor cells to NK cell killing. Our clinical study showed that gemcitabine treatment led to a transient increase of serum MICA levels in pancreatic cancer patients. The highest levels of serum sMICA were on day 3 after gemcitabine administration. Serum sMICA declined slightly thereafter, which could be due to tumoricidal effect of gemcitabine or due to the suppression of proteinase gene expression. Since soluble MICA/B can antagonize membrane-bound MICA/B-mediated antitumor immunity, the impact of genotoxic drug- or radiation-induced MICA/B expression in pancreatic tumor cells in a clinical setting remains unknown. Recent clinical trials revealed that pancreatic cancer patients treated with chemotherapy followed by immunization with a GM-CSF-transfected pancreatic cancer cell line developed a strong antitumor immunity and prolonged patient survival [38,39]. Combinational use of proteinase inhibitors that block MICA/B cleavage or use of chemotherapeutic drugs that can also suppress proteinase expression may further improve the therapeutic outcome of immunotherapy for pancreactic cancer.

## Conclusions

Our study has demonstrated that serum MICA levels and MICA/B expression in pancreatic tumor tissues are elevated. Radiation and genotoxic drugs are able to induce MICA/B expression in pancreatic cancer cells through the accumulation of uric acid. Gemcitabine therapy leads to increased serum MICA levels, probably as a result of increased MICA expression in tumor tissues.

## Abbreviation

**5-FU**: 5-fluorouracil; **Gem**: emcitabine; **IHC**: immunohistochemistry; **MHC**: major histocompatibility complex; **MICA/B**: major histocompatibility complex class I-related chain A and B; **MMP**: matrix metalloproteinase; **NK**: natural killer; **sMICA**: soluble MICA.

## Competing interests

The authors declare that they have no competing interests.

## Authors' contributions

All authors read and approved the final manuscript.

XX, GR, PG, J, & RAP: Conducted basic, pathological and clinical studies.

XX, VG, TS, HLK, JP, & RAP, conceived and executed the project, writing and/or critically reading the manuscript

## Pre-publication history

The pre-publication history for this paper can be accessed here:

http://www.biomedcentral.com/1471-2407/11/194/prepub
